# An Ensemble Learning Model for Detecting Soybean Seedling Emergence in UAV Imagery

**DOI:** 10.3390/s23156662

**Published:** 2023-07-25

**Authors:** Bo Zhang, Dehao Zhao

**Affiliations:** College of Information and Electrical Engineering, Heilongjiang Bayi Agricultural University, Daqing 163319, China

**Keywords:** emergence evaluation, unmanned aerial vehicle, imagery, ensemble learning model, growth stages, emergence proportion

## Abstract

Efficient detection and evaluation of soybean seedling emergence is an important measure for making field management decisions. However, there are many indicators related to emergence, and using multiple models to detect them separately makes data processing too slow to aid timely field management. In this study, we aimed to integrate several deep learning and image processing methods to build a model to evaluate multiple soybean seedling emergence information. An unmanned aerial vehicle (UAV) was used to acquire soybean seedling RGB images at emergence (VE), cotyledon (VC), and first node (V1) stages. The number of soybean seedlings that emerged was obtained by the seedling emergence detection module, and image datasets were constructed using the seedling automatic cutting module. The improved AlexNet was used as the backbone network of the growth stage discrimination module. The above modules were combined to calculate the emergence proportion in each stage and determine soybean seedlings emergence uniformity. The results show that the seedling emergence detection module was able to identify the number of soybean seedlings with an average accuracy of 99.92%, a R^2^ of 0.9784, a RMSE of 6.07, and a MAE of 5.60. The improved AlexNet was more lightweight, training time was reduced, the average accuracy was 99.07%, and the average loss was 0.0355. The model was validated in the field, and the error between predicted and real emergence proportions was up to 0.0775 and down to 0.0060. It provides an effective ensemble learning model for the detection and evaluation of soybean seedling emergence, which can provide a theoretical basis for making decisions on soybean field management and precision operations and has the potential to evaluate other crops emergence information.

## 1. Introduction

Smart agriculture promotes the deep integration of modern information technology and agricultural development; it helps to realize precise crop field management, improve crop production indicators, and contribute to sustainable agricultural development [1,2]. The application of optical imaging-based crop phenotype information collection platforms and data analysis technology is an important way to build crop growth models and obtain high-dimensional and rich phenotype datasets. It can provide the basis for quantitative decision-making and management in the agricultural development process [3,4]. Seedling stage is an important stage of crop growth; accurate and rapid access to crop emergence information helps to make field management decisions efficient and accurate crop growth assessments [5,6,7].

Satellite, UAV, and ground-based phenotyping platforms have been developed to improve the efficiency and accuracy of crop information detection in the field. The crops at the seedling stage are young, and the satellite platform is limited by the temporal and spatial resolution of data acquisition, and the revisit cycle is long and interfered with by the atmosphere, clouds, rain, and snow [8,9], so it is more difficult to apply it to seedling crop information detection. The images acquired by the ground platform have high resolution, but the imaging coverage is small, and the information collected is easily affected by ground conditions, which cannot meet the rapid detection of crop information in a large field area [10]. The UAV platform can obtain crop information at low altitude levels without direct contact with crops, regardless of field ground conditions, and with high resolution, providing an effective solution for crop phenotype analysis and assessment in the field [11,12]. Agricultural data come from a wide variety of sources, types, and structures, and artificial intelligence (AI) algorithms can quickly and automatically analyze agricultural data with these characteristics [13]. Under field conditions, manual counting of emergence information is tedious and time-consuming and is subject to serious human error. UAV platforms have improved the ability to effectively collect high-resolution images of crops at the field scale, and the combination of AI algorithms and UAVs provides conditions for research into crop emergence information detection.

The UAV image resolution is critical, and reducing or increasing resolution will affect crop feature extraction and recognition accuracy [14]. Resolution is closely related to flight altitude, with different crop coverage in the images at different flight altitudes, giving variability in the crop information extracted by UAV [15]. Dai et al. [16] achieved accurate extraction of information on cotton emergence, canopy cover, and growth uniformity using UAV RGB images and support vector machines (SVM), and they found that image resolution had a large impact on the model. Banerjee et al. [17] combined spectral and morphological information extracted from UAV multispectral images to effectively estimate wheat emergence using machine learning regression analysis, but the lower resolution posed difficulties in detecting the number of wheat emergences. The improved YOLOv4 proposed by Gao et al. [18] achieved accurate detection of maize numbers; they used depth-separable convolution and improved network structure to make the model more lightweight and reduce the number of model parameters, but the smaller range of acquired features for maize in the images made it less efficient. There will be some variation in the detection accuracy of the model for images acquired during different crop growth periods [19]. Du et al. [14] used UAV images to develop a mixed pixel decomposition model that can improve the accuracy of wheat basic seedling number inversion, which has high accuracy and applicability from the three-leaf stage to the overwintering stage. Jin et al. [20] effectively assessed the plant density of wheat at emergence from UAV images using SVM, but there were differences in recognition results for images acquired at different growth periods. High-quality datasets have been found in previous studies to be essential for model performance improvement. Liu et al. [21] considered that in order to make the model more accurate and efficient, data under different conditions could be collected extensively, and thus high-quality datasets could be built. The RiceNet model constructed by Bai et al. [22] estimated the location, plant size, and number of rice seedlings and achieved accurate rice planting management, but the datasets lacked diversity. Zaji et al. [23] implemented wheat spikelet localization and counting through a hybrid U-Net architecture, achieving good accuracy but with the problem of insufficient data quantity.

By summariaing the above research, it was found that the following issues need to be addressed: The image resolution has a large impact on the model, and the appropriate UAV flight height is closely related to the resolution. The lightweighting of the model is important for further research. The detection accuracy of the model varies for images acquired during different growing periods of the crop. Lack of diversity in the dataset and insufficient data sample size are common problems. Some of these problems can be solved by data augmentation, integration and optimization of algorithms, and determination of the optimal UAV flight height, while reduction of the total model parameters and creation of high-quality datasets are also essential and will be addressed in our study.

Therefore, our study innovatively proposes to construct an ensemble learning model. By improving, optimizing, and fusing multiple algorithms, we build a soybean emergence model based on UAV RGB imagery to achieve one-stop detection and evaluation of various soybean emergence information. The specific objectives included: (1) to construct an seedlings emergence detection module to obtain the number of soybean seedlings as well as to determine the optimal UAV sampling range by using UAV to collect images at different flight altitudes; (2) to evaluate the adaptability of two deep learning models, MobileNetV2 and AlexNet, for growth stages discrimination and explore the role of image enhancement in improving data quality and increasing model accuracy; and (3) to integrate the indicators to build an ensemble learning model to determine soybean seedling emergence uniformity by calculating emergence proportion, and guide intelligent field management and precision operations for soybeans during the seedling stage.

The rest of the paper is structured as follows: Section 2 contains the materials and methods used in this study. Section 3 analyses and discusses the results of the model and the application evaluation undertaken. Section 4 discusses the results of the study, the reasons for the experimental errors, and future prospects. Section 5 concludes the study.

## 2. Materials and Methods

### 2.1. Experimental Materials and Data Acquisition

**Figure 1:**
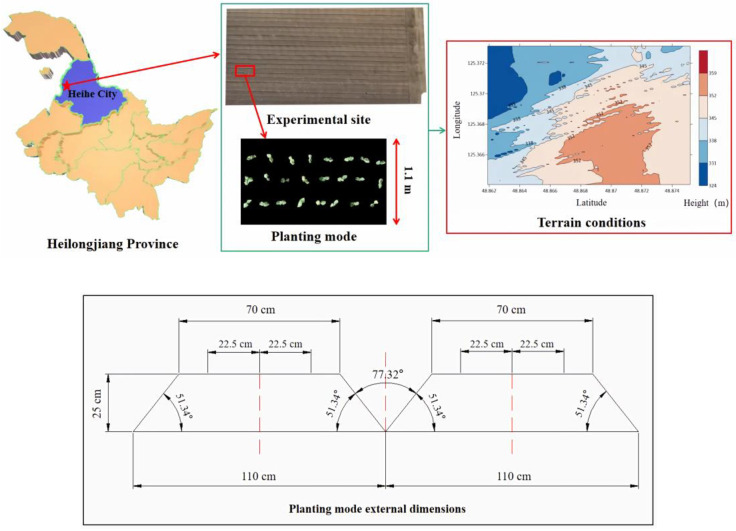
Schematic diagram of the experimental site and planting mode.

The experimental site was at North 15, Jianshan Farm, Heihe City, Heilongjiang Province, China (48°86′22″ N, 125°36′43″ E). A schematic diagram of the experimental site and planting mode is shown in Figure 1.

The overall view of the experimental site was obtained by image stitching after aerial photography by UAV; the total area of the experimental site was 54 hectares, and only part of the experimental area was shown here. In terrain conditions, the height lies between 324 m and 359 m above sea level. The main soybean varieties, Beidou 37 and Longken 3401, were selected and planted in three rows on a 1.1 m monopoly; each was planted in half of the field and was followed by the same number of plots. The marginal effect of monopolies can be fully exploited to increase soybean yields by increasing the number of soybean seedlings retained per unit area [24]. In the planting mode, the spacing between each two rows of soybeans is 22.5 cm, and the height of the monopoly is 25 cm.

**Figure 2:**
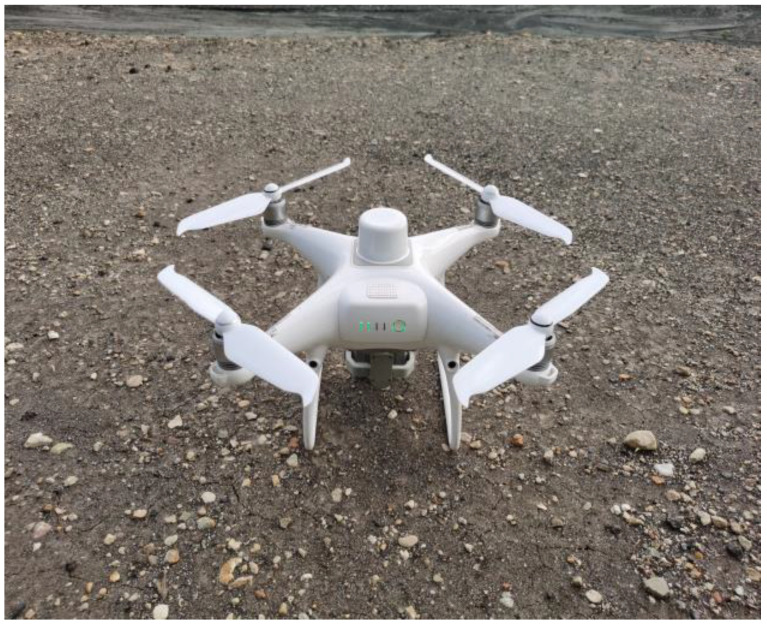
DJI P4 Multispectral UAV physical picture.

Using the DJI P4 Multispectral UAV (DJI, Shenzhen, Guangdong, China) as an image data acquisition platform. It integrates one RGB sensor and five multispectral sensors (R, G, B, RE, and NIR), each with 2 megapixels. Set the UAV flight parameters through DJI GS Pro software and shoot with the lens vertically down, original image resolution is 1600 × 1300 pixels. Figure 2 shows the UAV physical picture.

The UAV flight height is generally set artificially to ensure flight safety, no obstacles, and clear image acquisition; this mostly relies on rules of thumb for decision-making [15]. Consequently, when using UAV for soybean field image acquisition, the best flight height should be selected and sufficient resolution maintained to balance field coverage and algorithm recognition accuracy. The experiment was carried out at the early stage of soybean growth; if the flight height was too high, the algorithm would have difficulty recognizing the small soybeans in the images. If the flight height was too low, it would increase the flight time and affect efficiency. Therefore, in our study, three flight heights were used to acquire images: 3 m, 4 m, and 5 m, which were detected by the algorithm and then analyzed to determine the optimal UAV flight height.

Sixty monitoring sites were selected in the test plots for the experiment. During the soybean seedling stage, weeding, pest and disease control, fertilizer, and water retention are needed during the VE stage. The VC stage is important for weed control, and in the V1 stage, attention should be paid to weeds and pests. Thus, images acquired during these three stages (VE, VC, and V1) were used for the study. Soybeans were sown on the plot on 25 April and soybeans emerged from 16 May until 2 June when full V1 stage coverage was achieved. Soil temperature and cumulative temperature information was monitored from 26 April to 2 June and is shown in Table 1. The low temperatures in the early stages led to a late emergence of soybeans, which began to emerge rapidly when temperatures increased in the later stages. Considering the image quality, the images were chosen to be acquired in the absence of wind or breeze. In the soybean field environment, light and dark variations in light, complex weather conditions, and images acquired under complex backgrounds will affect the model’s performance. To reduce the impact of the above factors on model performance, a large number of soybean seedling images were acquired in a variety of environments, including sunny, cloudy, and rainy days and conditions with different light variations, as well as during different time periods in the morning, midday, and evening, respectively. Some of the images had V2 stages in them, and they were excluded. While the UAV completed the image acquisition task, the emergence status at that date at each monitored site was recorded manually.

### 2.2. Ensemble Learning-Based Soybean Seedling Emergence Detection Model

Combining the seedling emergence detection module, the seedling automatic cutting module, the growth stage discrimination module, and the emergence proportion calculation module, an ensemble learning model was constructed to realize one-stop detection of soybean seedling emergence. Ensemble learning trains several individual learners to complete the learning task through certain combination strategies, finally forming a strong learner. It can be applied to classification, regression, feature selection, and outlier detection.

#### 2.2.1. Seedling Emergence Detection Module

The seedling emergence detection module was constructed by Otsu with connected component analysis. The acquired soybean RGB images in the field were Otsu binarized and converted to color images, and then the number of soybean seedlings was calculated by the connected component analysis algorithm. Otsu is a classical adaptive thresholding algorithm for grayscale image thresholding segmentation, with the idea of finding the best threshold to maximize the inter-class variance of two classes after clustering [25]. As calculated using Equation (1) [26].

(1) $$\sigma^{2}\left(k\right)=w_{0}{\left(\mu -\mu_{0}\right)}^{2}+w_{1}{\left(\mu -\mu_{1}\right)}^{2}$$

where $\sigma^{2}$ is the inter-class variance, *k* is the threshold, the value of *k* when $\sigma^{2}$(*k*) takes the maximum value is the optimal threshold, *w*_0_ is the probability of occurrence of target image elements, *w*_1_ is the probability of occurrence of background image elements, *µ* is the image grayscale mean, *µ*_0_ is the target grayscale mean, and *µ*_1_ is the background grayscale mean.

The connected component analysis algorithm is generally used for binary images to find and label pixels that have the same pixel value and are adjacent to each other. In the experiment, the connected component analysis algorithm was adapted to soybean color image characteristics so that it could be directly applied to color images converted by Otsu binarization. Further, the soybean contours were detected, the contours found were traversed and marked by the minimum outer rectangle, and their positions and numbers were plotted to achieve emergence number detection. Each contiguous region was analyzed for one soybean to reduce redundant information and not contain parts of soybeans from other periods. Analysis using 8 contiguous regions, including the top, bottom, left, right, top-left, top-right, bottom-left, and bottom-right positions in the images in the immediate vicinity of 8 directions [27]. It was defined as Equation (2).

(2) $$N_{8}\left(P\right)=N_{4}\cup \left(x+1,y+1\right),\left(x+1,y-1\right),\left(x-1,y-1\right),\left(x-1,y+1\right)$$

where *N*_4_ is the 4 contiguous regions, *x* is the horizontal pixel coordinate, and *y* is the vertical pixel coordinate.

The coefficient of determination (*R*^2^), root mean square error (*RMSE*), and mean absolute error (*MAE*) were used as evaluation metrics (Equations (3), (4), and (5), respectively) [17]. *R*^2^ can reflect the fit degree of the trend line between the number of predicted soybean seedlings and real soybean seedlings. *RMSE* is the error dispersion degree between the number of predicted and real seedlings, representing the algorithm’s stability. *MAE* is the average error between the number of predicted and real seedlings, which indicates the algorithm’s accuracy.

(3) $$R^{2}=\frac{\sum i\left({\hat{y}}_{i}-\bar{y}\right)}{\sum i\left(y_{i}-\bar{y}\right)}$$

(4) $$RMSE=\sqrt{\frac{\sum i{\left({\hat{y}}_{i}-y_{i}\right)}^{2}}{N}}$$

(5) $$MAE=\frac{\sum i\left|{\hat{y}}_{i}-y_{i}\right|}{N}$$

where *i* is the *i*-th monitoring site, the range of *i* is 1–60, and the summation limit is 60. *y_i_* is the number of real seedlings in the i-th monitoring site, ${\hat{y}}_{i}$ is the number of predicted seedlings in the *i*-th monitoring site, $\bar{y}$ is the mean value of the number of seedlings in each monitoring site, and *N* is the number of monitoring points.

#### 2.2.2. Seedling Automatic Cutting Module

**Figure 3:**
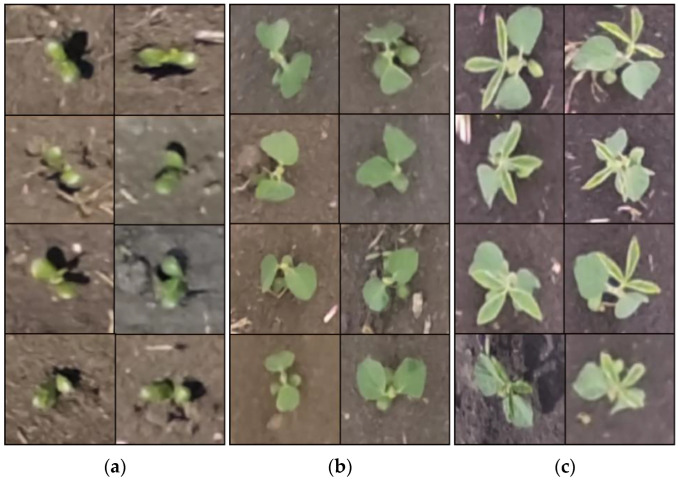
Images of soybean seedlings obtained by automatic cutting: (**a**) VE stage images; (**b**) VC stage images; (**c**) V1 stage images.

Five hundred images collected by UAV were chosen to identify and label soybean seedlings by the seedling emergence detection module, and the detected soybean seedlings were cut using an automatic cutting algorithm to build image datasets; some images are shown in Figure 3. The image size was adjusted to 255 × 255 × 3, and labels were made to distinguish between VE as “0”, VC as “1”, and V1 as “2”, for a total of 3000 images.

In order to obtain a neural network model with good performance and recognition accuracy that can detect crop information at different growth stages, more diverse and representative datasets were constructed using image enhancement techniques to train the model. Operations such as brightness, horizontal flip, saturation adjustment, random cropping, and scaling were performed on the original images to obtain the enhanced images, which were combined with the original images to form a new dataset. The imbalance of sample distribution in the datasets will lead to negative effects such as overfitting and difficulty in feature extraction. In order to ensure the model learning effect, 3000 images per stage in the datasets, or 9000 images in total, were used as the input layer of the deep learning model.

#### 2.2.3. Growth Stage Discrimination Module

The growth stage discrimination module was the main part of the soybean seedling emergence detection model, which classified the growth stage (VE, VC, or V1) by discriminating the soybean seedling images from the automatic cutting module. The model should be sufficiently lightweight to ensure its recognition performance and interference resistance in the field environment. Deep learning shows great potential in remote sensing monitoring of crop growth at the field scale [28], of which MobileNetV2 and AlexNet were selected to construct separate models for soybean seedling image characteristics and fully extract feature information.

**Figure 4:**
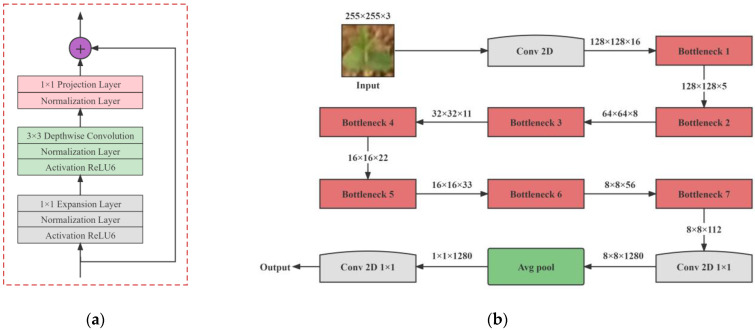
The architecture of MobileNetV2: (**a**) Inverted residual block structure; (**b**) MobileNetV2 network structure.

MobileNetV2 is a lightweight neural network proposed by Sandler et al. in 2018, retaining the deep separable convolution from the previous version and adding two modules for inverted residuals and linear bottlenecks [29]. The architecture of MobileNetV2 is shown in Figure 4.

Figure 4a is an inverted residual block structure. Inverted residual blocks adopt the structure of ascending and descending first, which can reduce the information loss, receive rich feature information, and then improve accuracy. Linear bottlenecks improve the nonlinear activation function ReLU6 after the layer with a smaller output dimension in the network to linear activation, which solves the problem of feature loss when compressing high-dimensional features to low-dimensional features [30]. Based on the soybean seedling image features, the MobileNetV2 network structure was designed as shown in Figure 4b, consisting of a two-dimensional convolutional layer, a bottleneck layer, a global average pooling layer, and a fully connected layer.

**Figure 5:**
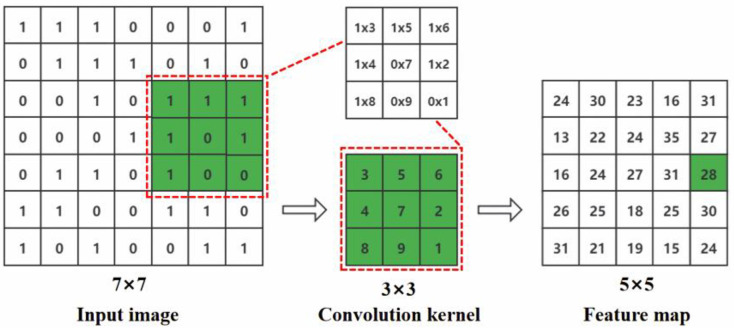
Convolution process.

AlexNet is a typical model in a convolutional neural network, consisting of five convolutional layers, three pooling layers, and three fully connected layers. The convolutional layer efficiently extracts deeper feature information from a small region of pixels in the images using a sliding calculation at stride 1; the convolution process is shown in Figure 5. Max-pooling reduces information loss by retaining the most significant image features, and the fully connected layer takes the main computational load and stores the final feature information of the model. AlexNet is normalized by LRN to suppress the feedback of smaller neurons and amplify the feedback of larger neurons, speed up the model convergence by the ReLU activation function, and introduce Dropout to prevent overfitting.

To evaluate the performance of the growth stage discrimination module and to ensure that soybean images are sufficiently diverse and representative to avoid the problem of weak model generalization due to single soybean image features, MobileNetV2 and AlexNet were compared for testing accuracy on the original image datasets (3000 images) and enhanced image datasets (9000 images), respectively. Dividing the training and testing sets by a ratio of 3:1, the distribution of image datasets is shown in Table 2.

Accuracy is one of the important metrics to evaluate the overall performance of a model, but for some specific tasks, the model needs to meet requirements for low parameter numbers, time efficiency, and stability. Using training time, average loss and average accuracy as evaluation metrics, the average loss, and average accuracy were calculated as shown in Equations (6) and (7).

(6) $$AL=\frac{e_{L}}{e}$$

(7) $$AA=\frac{e_{A}}{e}\times 100\%$$

where *AL* is the average loss, *AA* is the average accuracy, *e_L_* is the sum of loss after each epoch, *e_A_* is the sum of accuracy after each epoch, and *e* is the number of epochs.

#### 2.2.4. Emergence Proportion Calculation Module

Soybean emergence uniformity is highly significant and positively correlated with yield; soybeans that emerge later cannot compete with those that emerged earlier and do not grow vigorously, resulting in yield loss. Uneven soybean emergence includes the simultaneous presence of multiple growth stages of soybeans after emergence, failure to timely convert soybeans from the previous stage to the next growing stage within a reasonable period of time, small seedlings caught in large seedlings, and a low number of seedlings emerged, resulting in a poor emergence rate. The emergence proportion calculation module combined the output of the above modules: first, we obtained the overall number of soybean seedlings; second, we obtained the results of identifying the growth stages of all soybean seedlings; next, we calculated the soybean emergence proportion for each growth stage; and finally, we determined whether soybean seedling emergence was uniform. The emergence proportion was calculated as shown in Equation (8).

(8) $$EP=\frac{TP_{i}}{ST}$$

where *EP* is emergence proportion, *TP_i_* is the number of soybean seedlings that emerged at stage i, *i* is VE, VC or V1, and *ST* is overall number of soybean seedlings emerged.

To validate the model for practical effects in the field, a total of six dates—19 May, 20 May, 22 May, 23 May, 25 May, and 26 May—were selected between 16 May and 2 June, and 30 images acquired by UAV were used for emergence information detection on each date.

All models were in Python language, using Pycharm as a compiler. Deep learning was trained and tested using TensorFlow 2.5 and its built-in Keras module, applying Anaconda, CUDA, and cuDNN. The processor is an Inter(R) Core(TM)i5-1035G1 CPU @1.00GHz 1.19, running on Windows 10, 64 bit. The adapter parameters were displayed as Inter(R) UHD Graphics, NVIDIA GeForce MX350.

## 3. Results

### 3.1. Performance Evaluation of Seedling Emergence Detection Module

**Figure 6:**
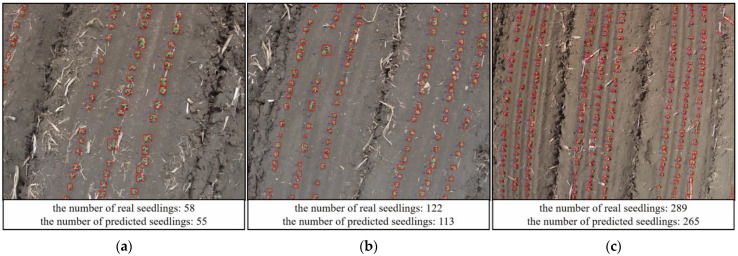
The detection results for three different flight heights: (**a**) The detection results of 3 m; (**b**) The detection results of 4 m; (**c**) The detection results of 5 m.

UAV images acquired at three different flight heights were examined with this module. On the one hand, the performance of the module was evaluated, and on the other hand, the optimal flight height for the UAV was determined. The detection results for three different flight heights were displayed visually, including the number of real seedlings and predicted seedlings, as shown in Figure 6. The results were highlighted by using red rectangular boxes to mark the target locations and blue to mark the number of seedlings.

**Figure 7:**
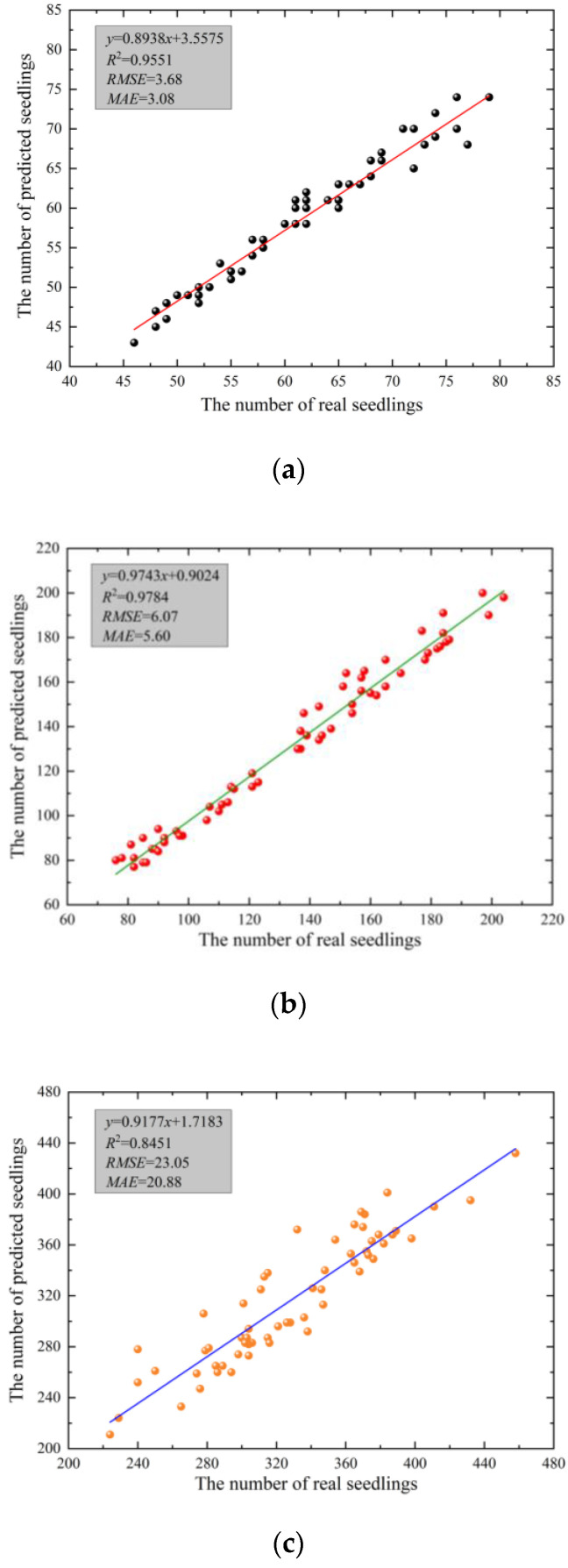
Comparison of the number of predicted and real seedlings: (**a**) Flight height of 3 m; (**b**) Flight height of 4 m; (**c**) Flight height of 5 m.

The monitored points were 60, and the images taken by UAV were predicted using the seedling emergence detection module and compared with the number of real seedlings measured manually. The results are shown in Figure 7.

Analysis of Figure 7 shows that when the UAV flight height was 3 m, in the results of the comparison between the number of predicted and real seedlings, R^2^ was 0.9551, RMSE was 3.68, and MAE was 3.08. When the flight height was 4 m, R^2^ was 0.9784, RMSE was 6.07, and MAE was 5.60. When the flight height was 5 m, R^2^ was 0.8451, RMSE was 23.05, and MAE was 20.88. The average accuracy of detecting images acquired at three flight heights was 95.64%, 99.92%, and 93.54%, respectively. When the flight heights were 3 m and 4 m, R^2^ was able to reach above 0.95, and the average accuracy was above 95%. Taken together, the R^2^ and average accuracy of the 4 m were higher, and although it was lower than the 3 m in two indexes, RMSE and MAE, it could obtain a larger range of field information and improve detection efficiency.

Morphological characteristics of soybean can vary between growing environments and between varieties. In order to determine whether the module can accurately detect other soybean varieties and soybean seedlings grown in different regions, we chose the Circular Agriculture Research Center of Guangdong Province (21°16′46″ N, 110°25′86″ E), which is geographically significantly different from Jianshan Farm, to carry out the experiment. Nine of the main planted soybean varieties in Heilongjiang Province were selected as the study objects: Beidou 37, Longken 3401, Mengdou 36, Heike 60, Jiuyan 13, Longken 310, Nenao 5, Heihe 43, and Heihe 52. The forms are round, broad, and lance-leaved, etc. A drip irrigation pipe was placed between each of the two rows of soybeans in the experiment, and drip irrigation was used to keep the soil moist and promote soybean growth.

**Figure 8:**
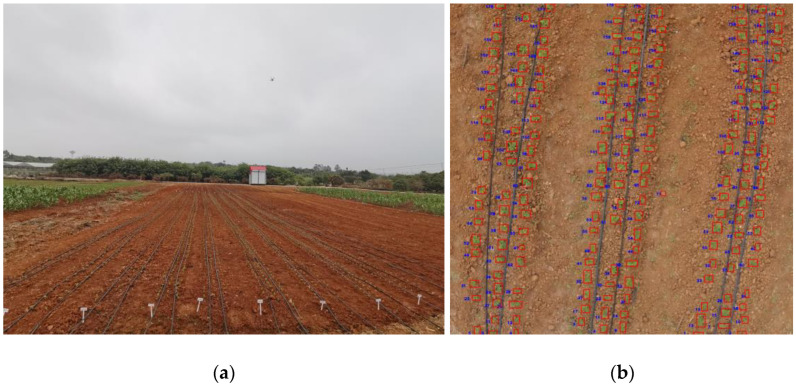
The overview of the experiment area and detection results: (**a**) The overview of the experiment area; (**b**) Detection results at 5 m.

By comparing the experiments at three different flight heights, we found that the best results were achieved at 5 m. The results of the inspection of 50 images show that the average recognition accuracy was 99.75% and the average recognition error was 0.25%, which produced some differences from Jianshan Farm. The overview of the experiment area and detection results are shown in Figure 8.

This was mainly due to the different spacing of the soybean seedlings at planting, which was 7.1 cm at Jianshan Farm and 16.5 cm here. The larger plant spacing reduced the problem of overlap between plants during soybean growth, making identification more accurate. This further demonstrates that the module we used does not produce errors depending on the soybean variety and that the module is reliable. This also provided us with ideas for further research, and for the identification of crops with different plant spacing, we should make several attempts to choose the best UAV flight height to obtain the data. This part of the experiment allowed the module’s performance to be verified, and all data used in the next experiments carried out were sourced from Jianshan Farm.

### 3.2. Performance Evaluation of Growth Stage Discrimination Module

**Figure 9:**
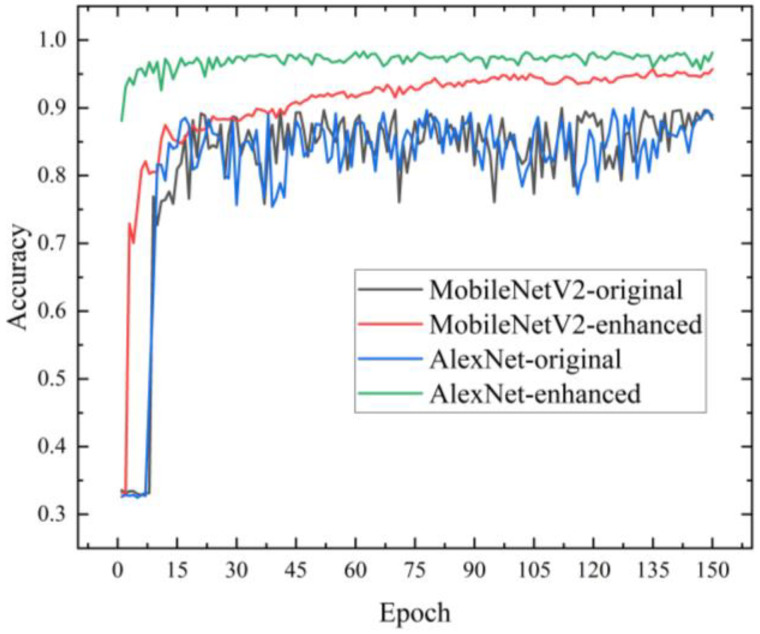
Trend of model test accuracy.

MobileNetV2 and AlexNet were analyzed to determine the best model to use as the backbone network for the growth stage discrimination module. The input data for this module were obtained from 4 m images by an automatic cutting module. Both models used Adam as the optimization algorithm with 150 iterations, and the trend of model test accuracy for the four cases is shown in Figure 9. From Equation (9), the model accuracy was defined.

(9) $$A=\frac{N_{C}}{N_{T}}$$

where *A* is the model accuracy, *N_C_* is the number of images correctly classified, and *N_T_* is the number of all images.

As can be seen from Figure 9, the test accuracy of both models fluctuated smoothly in magnitude compared to before enhancement. Image enhancement improved image quality, enriched the amount of information, and enhanced image interpretation and recognition. MobileNetV2 reached over 90% accuracy in 43 epochs, while AlexNet achieved it in 2 epochs and remained stabilized afterwards, indicating that AlexNet converges faster and more consistently. The evaluation of each performance of the model is available, as shown in Table 3.

Soybean seedling image datasets had the characteristics of a complex background and different morphological features of leaves. As shown in Table 3, facing this type of dataset, MobileNetV2 lacked scattered regions of interest and a single scale of feature extraction, so it caused poor recognition results. The optimal performance was achieved using AlexNet, with an average accuracy of 97.05%, an average loss of 0.0603, and a training time of 0.54 s/step. However, the model was not lightweight enough; the total number of parameters for the model was 29,751,811. Improvements can be made to the model to improve all its metrics.

**Figure 10:**
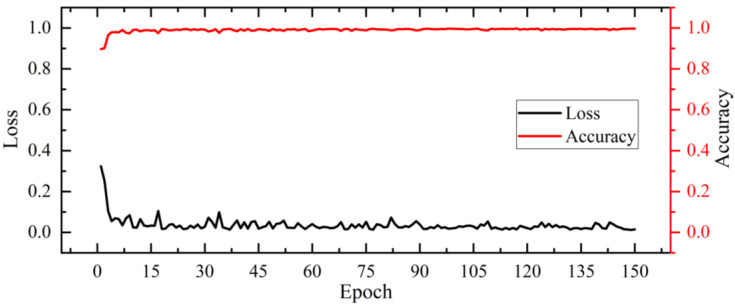
Trend of test accuracy and loss of improved AlexNet.

The number of total parameters for the model can be reduced by improving the network structure, which can reduce training time. The different sensitivity of the model to various parameters will lead to different experimental results, and the model’s accuracy and stability can be improved by finding the optimal combination of different parameters. Therefore, the fully connected layer structure of AlexNet was improved to 1024 and 256, and the model was tested under the optimal parameters, dropout was 0.6, batch size was 32, and learning rate was 0.0001. The trend of test accuracy and loss of improved AlexNet is shown in Figure 10.

Improved AlexNet was used to discriminate soybean growth stages, and the input images were 255 × 255 × 3. The convolutional kernel size kept getting smaller from 7 to 5 to 3, while the feature map sizes were also reduced by overlapping maximum pooling in half at layers 1, 2, and 5, and after the 5th convolutional layer, the image features were sufficiently refined. The final results show that the number of total parameters for the improved model was reduced to 6,140,419, the average accuracy was 99.07%, the classification accuracy was 98.99% in VE, 99.01% in VC, and 99.21% in V1, the average loss was 0.0355, and the training time was 0.53 s/step. The improved model not only achieved higher recognition accuracy but also had a smoother convergence process and fewer total parameters. At this point, we have obtained an optimal growth stage discrimination module that has been repeatedly trained so that different experiment areas and different soybean varieties do not affect the effectiveness of the module.

### 3.3. Model Actual Effect Verification

**Figure 11:**
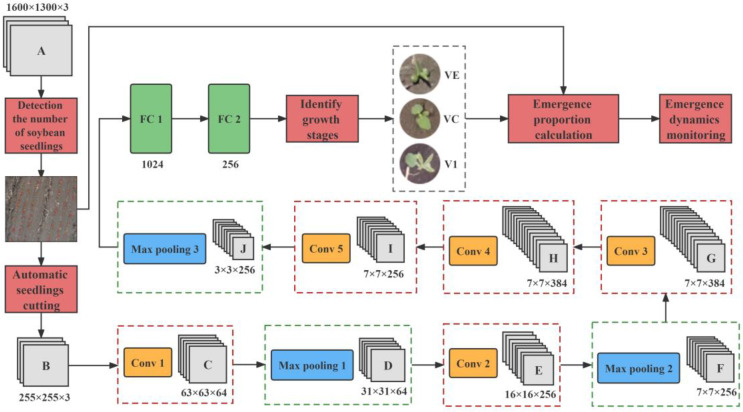
Ensemble learning-based soybean seedling emergence detection model.

By combining the outputs of the seedling emergence detection module, the seedling automatic cutting module, and the growth stage discrimination module, we were able to calculate the soybean seedling emergence proportion at each stage and construct the ensemble learning-based soybean seedling emergence detection model, as shown in Figure 11.

The actual effect of the ensemble model was verified by field experiments. Firstly, we were able to obtain the number of detected soybean seedlings. After classifying the growth stages of these detected seedlings, we were able to apply the model to calculate the proportion of seedlings at different growth stages in conjunction with Equation (8). The results of the predicted and real emergence proportions are shown in Table 4.

**Figure 12:**
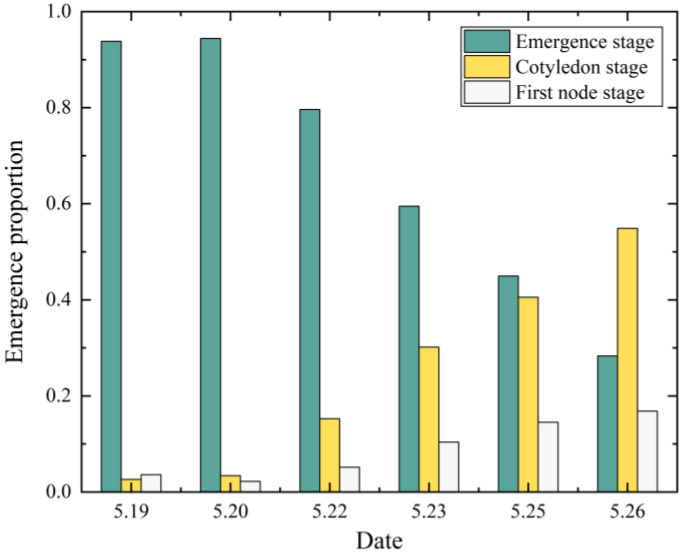
The dynamic process of soybean seedling emergence: The emergence stage is VE, the cotyledon stage is VC, and the first node stage is V1.

According to Table 4, the error between predicted and real emergence proportions was low, with a maximum of 0.0775 and a minimum of 0.0060. The dynamic process of soybean seedling emergence is shown in Figure 12. Combined with the agronomic requirements of soybean growth, 3–4 days after VE stage soybean seedlings were rapidly converted to VC stage, and on May 25, half of the soybean seedlings had been converted to VC stage, and a small amount of V1-stage soybean seedlings appeared. The number of VC-stage soybean seedlings had surpassed VE-stage by May 26. When VC-stage soybean seedlings appeared, the soybean growth stages in the field were mostly two stages (VE, VC, or VC, V1) or three stages coexisting (VE, VC, and V1). The overall emergence was more uniform, in line with the dynamic pattern of soybean growth over time.

## 4. Discussion

### 4.1. Analysis Based on Other Crops in Emergence

It can be seen that the module built in our study performs well overall but still lacks in some aspects. Sun et al. [31] constructed the Wheat Ear Counting Network (WECnet) using UAV images for accurate counting and density estimation of wheat. WECnet achieved R^2^, RMSE, and MAE of 0.95, 6.1, and 4.78 on the global wheat dataset, and R^2^ of 0.886 in UAV image counting, with an error rate of 0.23%. The model constructed in our study outperforms WECnet in terms of R^2^, RMSE, and error rate, but MAE is not as good as WECnet. Feng et al. [6] estimated the number of cottons (R^2^ = 0.95) and canopy size (R^2^ = 0.93) using ResNet18; although R^2^ was not better than the model in our study, it had a higher image resolution and UAV flight height, which was superior in terms of acquisition efficiency. Some scholars have estimated the number of maizes under different planting densities using U-Net with the highest R^2^ of 0.95 [32]; using ResNet18 to estimate maize emergence uniformity, the accuracy was 0.97, 0.73, and 0.95 in three different metrics; in our study, the accuracy of over 0.90 was achieved in each condition; however, maps were created to visualize the emergence in their study [33]. In our study, by designing three sampling ranges to balance the relationship between UAV flight height and recognition accuracy, we can provide ideas for efficient detection of crop emergence.

By comparing the results obtained by some scholars in similar studies, the methodology, experimental design, and sensors used in our study can be useful for conducting similar studies at present. Specifically, the RGB sensors demonstrate a strong capability in crop emergence information monitoring; although they cannot achieve crop growth monitoring by analyzing the spectral information of the crop as spectral sensors can, the RGB sensors can be analyzed by extracting features such as color, texture, and shape of the crop from the image. We chose to conduct experiments at two experimental sites with different geographical conditions and acquire images in different field environments. It is important to take care of these experimental designs when conducting similar studies, as it is important for the model to be applied in more scenarios and to demonstrate the model’s ability to generalize.

### 4.2. Analysis Based on Model-Generated Errors

There were problems with individual soybean seedlings being misclassified as other stages in the model prediction process, as shown on May 19 and May 20, when the actual soybean growth in the field did not reach the V1 stage. Thus, the causes of the error were analyzed: (1) The experimental site was preceded by corn; the presence of some corn stalks in the field, smooth stalks that reflect light more strongly and are similar in color to soybean seedlings, was mistakenly detected as soybean seedlings and misjudged for a certain stage. (2) Strong light caused soybean seedlings to appear white and could not be fully extracted and identified, resulting in missed detection. (3) Sowing spacing was small, and some of the vigorously growing soybean seedlings were interconnected, detected as one, and misclassified as other species during classification. (4) Weeding measures were taken in a timely manner during field management, with fewer or no weeds appearing in the early stages. The errors caused by weeds were reduced by setting thresholds, but some weeds with similar morphology to soybean seedlings were easily misclassified as soybean seedlings.

There was some variability in the model outputs due to the different ways in which soybean seedlings are composed of characteristics at different growth stages. Our model enabled the identification of VE, VC, and V1 stages, but control measures are still needed to ensure efficient and high-quality soybean growth in subsequent growth stages such as V2 and V3. As soybean seedlings grow progressively more vigorously, further improvement or optimization of the model and attempts to combine it with other algorithms will be needed in order not to affect the recognition results. For the construction of this module, the differences in detection accuracy between stages were reduced, verifying the power of deep learning in crop information detection, demonstrating the important role of image enhancement, and providing a method for other crops to carry out information detection in different growth stages.

### 4.3. Summary and Analysis of Future Prospects

For these detection models, the complexity of the structure, the memory footprint, and the low number of images in the dataset will affect their application in more scenarios in the future. The present detection model is processed and analyzed on the computer after UAVs collect images from the field. The trend is to deploy it on mobile devices so that collection can be performed anytime and on demand, data can be uploaded instantly, and statistical analysis results can be presented immediately. This requires the model to be sufficiently lightweight and reduce memory consumption. Therefore, some advanced techniques, such as network pruning and weight quantization, can be considered to reduce the model’s complexity and memory footprint [34,35]. Tang et al. [36] proposed a new automatic pruning method called sparse connectivity learning (SCL), which resulted in a significant reduction in the number of model operations and computational resources. Hu et al. [37] proposed a novel channel pruning method via class-aware trace ratio optimization (CATRO) to reduce the computational burden and accelerate model inference. If the number of images or other signaling information in the dataset is not sufficient for model training, the use of transfer learning techniques can be considered [38,39]. Zheng et al. [40] proposed a method of transferable feature learning and instance-level adaptation to improve the generalization ability of deep neural networks so as to mitigate the domain shift challenge for cross-domain visual recognition.

The YOLO series algorithms have shown strong capability in detecting the emergence of crop seedlings. Compared to the YOLO series algorithms, the ensemble learning model constructed in our research has been improved and optimized to make the model lighter, reducing computational time and resources. By combining different base models, the ensemble model can reduce the risk of overfitting a single model and improve the overall generalization capability [41]. This has been confirmed in previous studies carried out by many scholars. Liao et al. [42] presented a global and local ensemble network for objects in aerial images; the strengths and weaknesses of Yolov5 and CenterNet were fully considered, and the accuracy of the ensemble model constructed was significantly improved over the previous model. However, as mentioned earlier, for aerial images, different flight altitudes may lead to different detection results for the model, which is something missing in their study. Usha et al. [43] integrated the faster R-CNN and YOLO models to achieve vehicle detection and traffic density estimation by constructing EnsembleNet. The accuracy of EnsembleNet, faster R-CNN, and YOLO was 98%, 97.5%, and 95.8%, respectively. EnsembleNet achieved higher detection accuracy than the single model, but in terms of processing time, EnsembleNet took more time than the other two models. In the experiments, it was found that the execution time of the soybean emergence ensemble model was reduced by 18% compared to the cumulative time of multiple single models, allowing for increased efficiency while maintaining recognition accuracy. The issue of the execution time of the ensemble model was also illustrated in the study by Hanse et al. [44]. They constructed EnsemblePigDet for pig detection, and the execution time of the ensemble model was slower than that of the single model. This issue was fully considered at the initial stage of our research, and the complex structure of the single model was improved during the integration of multiple models, enabling the overall execution time of the ensemble model to be reduced. To conclude, in future research on the application of ensemble models, in addition to considering factors such as accuracy and stability, we should focus on how to reduce the execution time of the models, which will enable us to improve efficiency and carry out more accurate operations in future applications.

### 4.4. Model Scalability and Use of Other Sensors

In our study, the detection of emergence information for soybean seedlings using RGB sensors demonstrates that RGB sensors are capable of this task. However, with the continuous development of precision agriculture and various sensors, there are already many types of sensors that are widely used in agriculture, such as spectral sensors. UAV spectral imaging technology is a fast and new type of farmland environmental monitoring technology that can quickly obtain instant spectral images of farmland crops and obtain the growth information of field crops. Some scholars have used UAV spectral data combined with algorithms to achieve effective estimation of soybean yield [45]. In the future, we can consider other sensor data, such as multispectral or hyperspectral images. And, in order for the model to accommodate information from different sensors, it may involve adapting the model structure and parameters or even creating a sensor fusion model that can take advantage of RGB and multispectral/hyperspectral sensors to provide a more comprehensive and accurate analysis.

In addition to considering how models can be improved and optimized, and the causes of errors, cost may also be a key consideration if a long-term study is undertaken. The cost of maintaining the UAVs and the cost of deploying and maintaining an ensemble of models can be much higher than using a single model. This includes computational costs and the costs associated with data storage and processing. A cost-benefit analysis will help elucidate the possibility of implementing the proposed approach in a day-to-day working environment.

## 5. Conclusions

This study developed an ensemble learning model for assessing soybean emergence information using UAV RGB images. The model can be used to detect soybean numbers, identify soybean growth periods, and calculate soybean emergence proportions in each period.

UAVs carrying different sensors need to be adjusted to the appropriate flight height to be able to acquire useful images for analysis. The focus of our study was to explore the optimal flight height for RGB sensors carried by UAVs. Three flight heights were selected to capture images in the experiment and identified using our proposed algorithm, which provides ideas for the selection of the optimal flight height for obtaining seedling crop information using UAV RGB sensors. Not only was the optimal UAV sampling range determined, but the influence of image resolution on the accuracy of soybean feature extraction and recognition was reduced, while the efficiency of UAV image acquisition was improved. In the image information acquired by the UAV, there may be noise in the data due to wrong data collection and inaccurate GPS coordinates, which will affect the performance of the model. We have established a high-quality dataset through image enhancement technology, which effectively enhances the crop feature information in the images, improves the generalization ability and efficiency of the model, and makes the model more stable. In real-world agricultural scenarios, the value of such systems would be significantly increased by providing real-time data to allow immediate field management decisions, in which model lightweighting has an important role. By improving and optimizing AlexNet in our study, the total parameters of the model were reduced by more than four times. The use of these treatments in combination with multiple algorithms allows the model to be lightweight while maintaining a certain level of accuracy in complex environments and areas with different geographic conditions, which can provide a basis for real-time data processing in the future.

The overall assessment of soybean field emergence information using the model shows that the error between the predicted and the real emergence proportion was small, and the uniformity of soybean emergence could be effectively determined. The model can be used as a powerful tool for comprehensively assessing soybean field emergence, helping agricultural workers make better field management decisions, and providing ideas for applying deep learning models to real-time processing of field data.

## Figures and Tables

**Table 1:** Soil temperature averages and cumulative temperature information.

Date	Soil Temperature Averages	Cumulative Temperature
4.26–5.15	9.75 °C	154.3 °C
5.16–6.2	16.1 °C	265 °C

**Table 2:** Distribution of image datasets.

Image Types	Growth Stages	Labels	Training Sets Numbers	Testing Sets Numbers
Original images	VE	0	750	250
	VC	1	750	250
	V1	2	750	250
Enhanced images	VE	0	2250	750
	VC	1	2250	750
	V1	2	2250	750

**Table 3:** Model performance evaluation.

Datasets	Training Time	Average Loss	Average Accuracy
MobileNetV2-original	0.79 s/step	0.1048	82.29%
MobileNetV2-enhanced	0.65 s/step	0.0762	90.71%
AlexNet-original	0.67 s/step	0.0904	81.87%
AlexNet-enhanced	0.54 s/step	0.0603	97.05%

**Table 4:** The results of predicted and real emergence proportions.

Date	Predicted Emergence Proportion			Real Emergence Proportion			Error		
	VE	VC	V1	VE	VC	V1	VE	VC	V1
5.19	0.9380	0.0261	0.0359	0.9830	0.0170	0	0.0450	0.0091	0.0359
5.20	0.9440	0.0339	0.0221	0.9568	0.0432	0	0.0128	0.0093	0.0221
5.22	0.7960	0.1524	0.0516	0.8548	0.1126	0.0326	0.0588	0.0398	0.0190
5.23	0.5948	0.3015	0.1037	0.6723	0.2659	0.0618	0.0775	0.0356	0.0419
5.25	0.4495	0.4054	0.1451	0.4892	0.4163	0.0945	0.0397	0.0109	0.0506
5.26	0.2830	0.5487	0.1683	0.3275	0.5547	0.1178	0.0445	0.0060	0.0505

## Data Availability

Not applicable.
